# Efficient solution for finding Hamilton cycles in undirected graphs

**DOI:** 10.1186/s40064-016-2746-8

**Published:** 2016-07-28

**Authors:** Wadee Alhalabi, Omar Kitanneh, Amira Alharbi, Zain Balfakih, Akila Sarirete

**Affiliations:** 1 Faculty of Computing and Information Technology, King Abdulaziz University, Jeddah, Saudi Arabia; 2 College of Engineering, Effat University, Jeddah, Saudi Arabia

**Keywords:** Undirected graphs, Hamiltonian cycle, Necessary condition, Set partition

## Abstract

The Hamilton cycle problem is closely related to a series of famous problems and puzzles (traveling salesman problem, Icosian game) and, due to the fact that it is NP-complete, it was extensively studied with different algorithms to solve it. The most efficient algorithm is not known. In this paper, a necessary condition for an arbitrary un-directed graph to have Hamilton cycle is proposed. Based on this condition, a mathematical solution for this problem is developed and several proofs and an algorithmic approach are introduced. The algorithm is successfully implemented on many Hamiltonian and non-Hamiltonian graphs. This provides a new effective approach to solve a problem that is fundamental in graph theory and can influence the manner in which the existing applications are used and improved.

## Introduction and preliminaries

The Hamiltonian cycle (HC) problem has many applications such as time scheduling, the choice of travel routes and network topology (Bollobas et al. 1987; Akhmedov and Winter 2014). Therefore, resolving the HC is an important problem in graph theory and computer science as well (Pak and Radoičić 2009). It is known to be in the class of NP-complete problems and consequently, determining if a graph is Hamiltonian, using the current algorithms, if it has a high time complexity. The difficulty of finding HC increases exponentially with the problem size. Also, there is an algorithm for solving the HC problem with polynomial expected running time (Bollobas et al. 1987).

A Hamiltonian path is a path in an undirected graph that visits each vertex exactly once. A Hamiltonian cycle is the cycle that visits each vertex once. A Hamiltonian graph is a graph that has a Hamiltonian cycle (Hertel 2004).

Due to their similarities, the problem of an HC is usually compared with Euler’s problem, but solving them is very different. There exists a very elegant, necessary and sufficient condition for a graph to have Euler Cycles. Also, literature presents many solutions that generate efficient algorithms for finding Euler Cycles. Unfortunately, there is a lack of solutions for the Hamilton problem. In addition to the author’ knowledge, there is no research indicating a necessary and sufficient condition for a graph to have a HC. Some of the theorems provide finding sufficient conditions for a graph to be Hamiltonian [such as Ore’s (1961) and Dirac’s (1952) theorems]. Nevertheless, the conditions in those theorems are complicated, and they are not applicable in many situations.

In this context, this paper introduces necessary conditions for a graph to have HC. This condition leads to build an efficient algorithm to find HC. The proposed algorithm depends on a numerical term which we call surplus edges.

### **Definition 1**

If *G*(*V*, *E*) is an undirected graph and *v* ∊ *V* is a vertex in *G*, then we define the surplus of ***v***, denoted by *sur*(*v*), to be *sur*(*v*) = deg (*v*) − 2. The surplus of the vertex *v* represents the number of edges that are incident to *v* and that must be removed so that the degree of ***v*** is 2.

In this paper, another new term used is: “n-factor graphs”. It is an extension to the family of Hamiltonian graphs.

### **Definition 2**

An undirected graph *G*(*V*, *E*) is n-Factor graph if, and only if there exists, a positive integer *n* and *G*_1_(*V*_1_, *E*_1_), *G*_2_(*V*_2_, *E*_2_),…, *G*_*n*_(*V*_*n*_, *E*_*n*_) cycles and sub-graphs of *G* with {*V*_1_, *V*_2_,…, *V*_*n*_} is a partition for *V*. Moreover, the set {*G*_1_, *G*_2_,…, *G*_*n*_} is called a factorized representation of *G* and the *G*_*i*_’s are called the factors of the representation. The *n* is the number of factors.

The remainder of this paper is as follows. In “Literature review” section, a short literature review related to the problem of HC is done. “Deletion and n-factor graphs” section tackles the problem of deletion and n-Factor Graphs, the basis of the algorithm proposed being detailed step by step. In “Results and discussion” section, the results of different case studies (examples) were given. The last section concludes the paper.

## Literature review

HC plays an important role in many areas including graph theory, algorithm design, and computational complexity. It has been studied widely in the past 100 years (Ibarra 2009). Many sufficient conditions for Hamiltonicity have been discovered from which two of the most notable are Dirac’s sufficient condition (Dirac 1952) and Ore’s theorems (Ore 1961).

### **Dirac’s theorem**

A simple graph with *n* vertices in which each vertex has degree at least $\left\lceil {n/2} \right\rceil$ has a Hamiltonian cycle.

### **Ore’s theorem**

A simple graph with *n* vertices in which the sum of the degrees of any two non-adjacent vertices is greater than or equal to *n* has a Hamiltonian cycle.

An algorithm for finding a HC in a proper interval graph in *O*(*m* + *n*) time is presented by Ibarra (2009) where m is the number of edges and n is the number of vertices in the graph. The algorithm is simpler and shorter than the previous versions. An interval graph is a graph where each vertex can be assigned an interval on the real line so that two vertices are adjacent if, and only if, their assigned intervals intersect; such an assignment is an interval representation. A proper interval graph is an interval graph with an interval representation where no interval is properly contained in another.

In another work, Nivasch (2003) tested two algorithms to determine their performance for practical problems. The two algorithms used are Ham and SemiHam. Ham was introduced by Bollobas et al. (1987), and it is heuristic polynomial-time algorithm for finding Hamiltonian cycles in random graphs with high probability.

In order to improve the Hamiltonian Cycle function of the Combinatorica, Csehi and Toth (2011) proposed an alternative solution for finding HC by testing if a HC exists. This is performed by checking the bi-connectivity of the graph and other conditions such as those described in the theorems of Dirac (1952), Ore (1960), Posa (1962) and Lawler et al. (1985).

A similar approach for finding HC in directed graphs is proposed by Christofides et al. (2012). It uses several known criteria for the existence of HC and provides algorithmic proof for, and oriented analogue of, Dirac theorem and an approximate version of Chvatal theorem.

Other researchers focused on specific structures. Liu and Wang (2014) demonstrated that, in a hypercube with a set of faulty edges, there is a fault free HC if two conditions are satisfied: the degree of every vertex is at least two and there are no pairs of adjacent vertices (with degree two) in a 4 cycle. In a similar study, Hao et al. (2014) derived that in a balanced hypercube, for any fault free edge exists a fault free HC containing it, and having *2n*-*2* faulty edges.

## Deletion and n-factor graphs

In order to find a HC, the edges that form it must be chosen so that any extra edges are avoided. In other words, the edges that do not belong to this cycle must be deleted. Definition 3 below introduces the primary way of deleting such edges.

### **Definition 3**

A deletion of a graph *G* is a random way of removing edges that are not incident to vertices with zero surpluses until we cannot delete more edges. The edges that are deleted are called surplus edges, and the deletion process is denoted by *D*.

This process is expressed in the Algorithm 1 below:
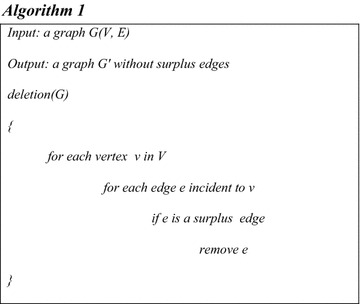


### **Definition 4**

In a deletion for a graph *G (V,E)*, an alternate edge path from vertex *u* to vertex *v* is defined to be a path in the graph G which is labeled in the deletion by *X,O,X,*… Or *O,X,O,X,…* In this case, “X” labels the removed edges, while “O” labels the non-removed edges.

Depending on the number and type of edges, a specific terminology is used:

- If the number of edges in an alternate edge path is odd, then the path is called an “odd alternate edge path”.
- If the odd alternate edge path starts with *O* (and so ends with *O* because its length is odd), then it is called a “*Destroyer*”.
- If the number of edges in an alternate edge path is even, then the path is called an “*even alternate edge path*”.
- If the even alternate edge path is a cycle from the vertex to itself, then it is called a “*Connector*”.

### **Definition 5**

The inverse of an alternate edge path: *XOXO…n*-times is the same path but with reflecting the *X*’s and the *O*’s, that is: *OXOX…n*-times.

### **Definition 6**

In a deletion *D* for a graph *G* (*V*,*E*), the set of vertices remained with surplus >0 (degree > 2) is called the Remaining Set induced by *D*, it is denoted by S_D_.

The following example illustrates the deletion process.

### *Example 1*

Consider the following graph G.

**Fig. 1:**
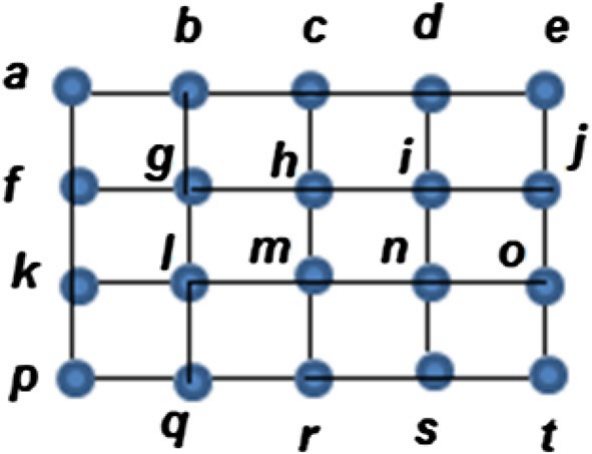
Initial *G* graph

**Fig. 2:**
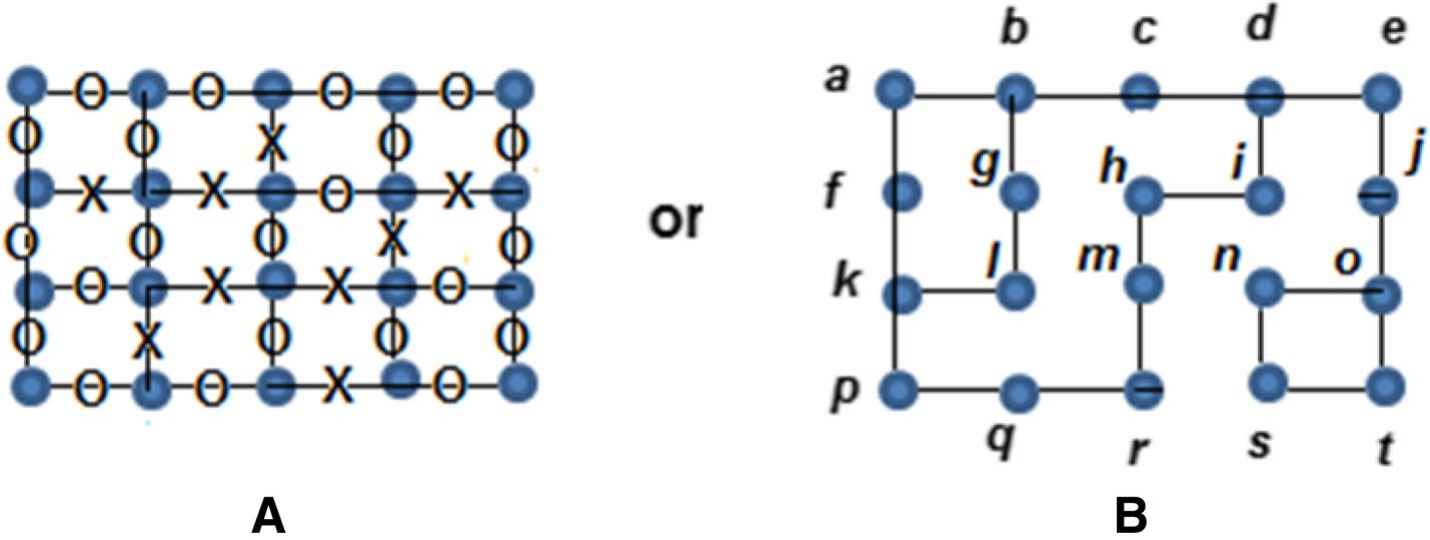
The reduced form of *G* in Fig. 1 after removing the edges mentioned in Example 1: **A** the graph with the XOX notation and **B** the graph without the XOX notation

According to definition 2, if the edges {c,h}, {f,g}, {g,h}, {i,j}, {i,j}, {l,m}, {m,n}, {l,q} and {r,s} are removed or labeled with *X*, while, the other edges are labeled with *O*, then the graph in Fig. 1 becomes the graph in Fig. 2, where Fig. 2A shows the graph with the XOX notation. Figure 2B shows the clean version of the graph without the XOX notation:

Now, no more edges can be removed because each edge is incident with at least one vertex with degree two (zero surplus).

Although no more edges can be removed, the graph still has some vertices of degree higher than two. Therefore, the objective becomes a refinement of the random deletion in order to reach a connected graph with degree two for each vertex. In order to perform such a task, Definitions 2 and 3 are considered and their corresponding algorithms being the following:
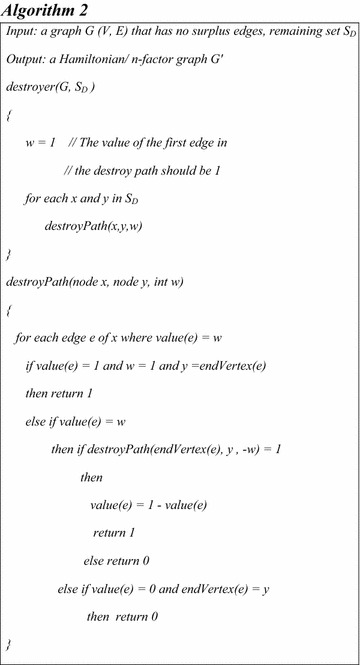


By analyzing the deletion from Example 1 leading to Fig. 2, the following aspects can be observed:

- Initially, a random deletion was applied so that the degree of every vertex is 2 (as discussed in Definition 3).
- The path f,g,l,m,h is an even alternate edge path with labeling *XOXO*,
- The path b,g,f,k is a destroyer with labeling P: *OXO,* according to Definition 4.
- According to Definition 5, the inverse of the destroyer b,g,f,k is b,g,f,k with labeling P^−1^: *XOX*.
- The remaining set of our deletion is *S*_*D*_ = {b,d,k,o}.

The following theorem provides a necessary and sufficient condition for a graph to be an n-factor graph.

### **Theorem 1**

*A graphG(V,* *E) is an n*-*factor graph if and only, if in any deletion D with a remaining set SD the following condition is satisfied:* “∀*u* ∈ *S*_*D*_∃*v* ∈ *S*_*D*_*such that there exists a destroyer from u to v*”.

### *Proof*

(←) If *S*_*D*_ = *φ*, then there is nothing to prove. If not, that is, if *S*_*D*_ ≠ *φ*, then by given ∀*u* ∈ SD∃*v* ∈ *S*_*D*_ and a destroyer, say P: u, *O*, × 1, *X*, x2, *O*,…, *X*, *x*_*n*_, *O*, *v*, where *x*_*i*_, are the vertices between u and v through which the destroyer P passes and *i* = 1,2,…*n*. By reflecting the destroyer P, its inverse is *P*^−1^: *u*, *X*, *x*_1_, *O*, *x*_2_, *X*,…, *O*, *x*_*n*_, *X*, *v*. This process reduces with one the degree of *u* and *v* without affecting the degrees of any *x*_*i*_, and this is because, simultaneously, one edge is added to each vertex *x*_*i*_ and one edge is removed from each vertex *x*_*i*_. Finally, if this process is repeated for the other vertices in SD, then some edges can be deleted so that the degree of each vertex in the resulting graph is 2. As a result, it is *S*_*D*_ = *ϕ*. This new graph is a factorized representation for graph *G*.

(→) If a graph G is n-factor, then it has already a factorized representation with n components. Therefore, a random deletion for G produces a graph that can be transformed into this representation by removing one edge (X) or removing two edges and adding one (XOX), and so on. This means that after that random deletion, the destroyers ((O) or (OXO) and so on) are reflected between the elements in the produced graph. It is important to mention here that the total sum of surplus edges is always an even number, and this can be seen through the well-known Handshaking Theorem that interprets the third equality in Eq. (1):

1 $$\sum\limits_{x \in V} {sur(x)} = \sum\limits_{x \in V} {\left( {\deg (x) - 2} \right)} = \sum\limits_{x \in V} {\deg (x) - } \sum\limits_{x \in V} 2 = 2\left( {\left| E \right| - \left| V \right|} \right),$$

where |*E*| is the number of edges and |*V*| is the number of vertices.

### *Example 2*

**Fig. 3:**
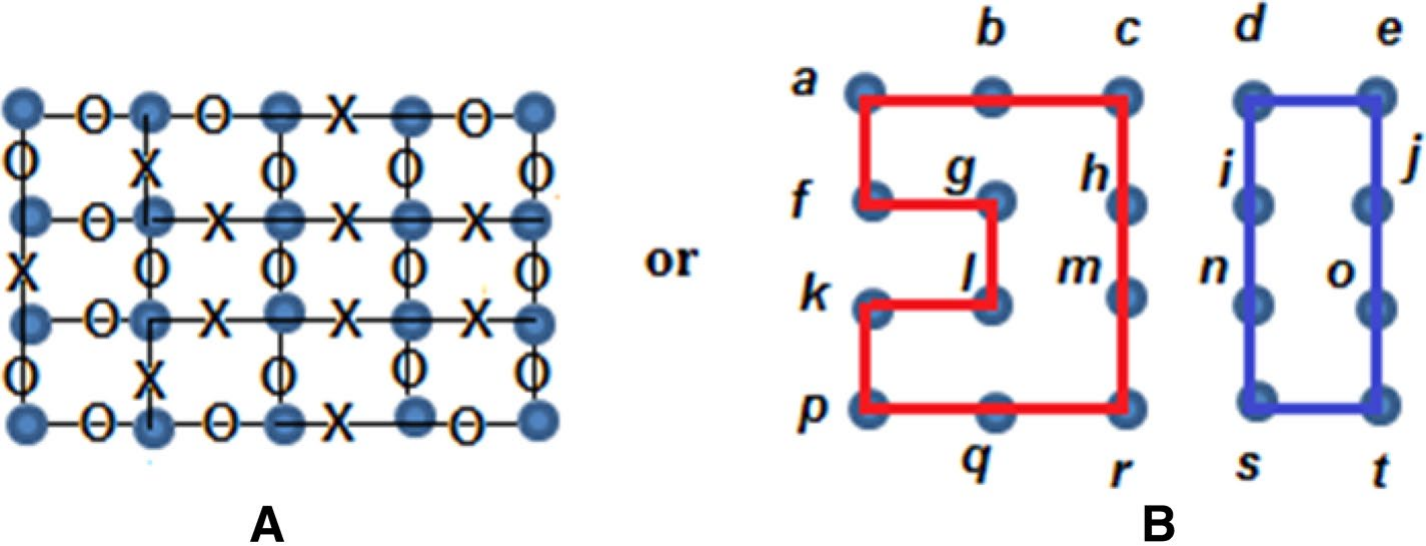
Applying the Destroyer Algorithm: **A** the XOX notation and **B** the clean version of the graph

Based on Example 1, it will be shown that $G$is an *n*-factor graph because $G$ satisfies Theorem 1. In order to demonstrate this, consider the remaining set S_D_ = {b,d,k,o}. For instance, the destroyer $P{:}\,b\underline{,O} ,g,\underline{X} ,f,\underline{O} ,k$ joins the vertices *b* and *k*. Also, the vertices d and o are joined by the destroyer $Q{:}\,d\underline{,O} ,c,\underline{X} ,h,\underline{O} ,i,\underline{X} ,n,\underline{O} ,o$. If $P$ and $Q$ are reflected, then $P^{ - 1}{:}\,b\underline{,X} ,g,\underline{O} ,f,\underline{X} ,k$ and $Q^{ - 1}{:}\,d\underline{,X} ,c,\underline{O} ,h,\underline{X} ,i,\underline{O} ,n,\underline{X} ,o$. These two reflections transform the graph from Fig. 2 to the graph from Fig. 3.

Figure 3, it can be observed that $C_{1}$: (g,h,m,l,g), $C_{2}$: (q,l,g,h,m,n,s,r,q), $C_{3}$: (m,n,s,r,m),$C_{4}$: (h,i,n,m,h), $C_{5}$: (c,d,i,h,c) and $C_{6}$: (i,j,o,n,i) are connectors, and there could be other connectors.

Reflecting any connector in an *n*-factor graph will transform it into an *m*-factor graph, where *m* is not necessarily to be equal to *n*. Therefore, the objective is to find the set of reflections that lead to the 1-factor graph, which is the Hamilton cycle of the original graph.

The concept behind reflecting a connector in a factorized representation is just adding *k*_*i*_ and removing *k*_*i*_ edges from each vertex *v*_*i*_. This means that reflecting connectors preserves the degree of each vertex but change the status of the graph from a representation to another.

The process of connecting an *n*-factor graph is expressed in Algorithm 3.
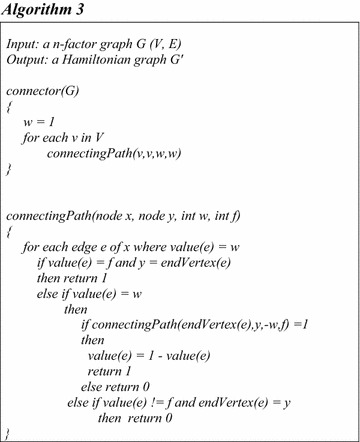


**Fig. 4:**
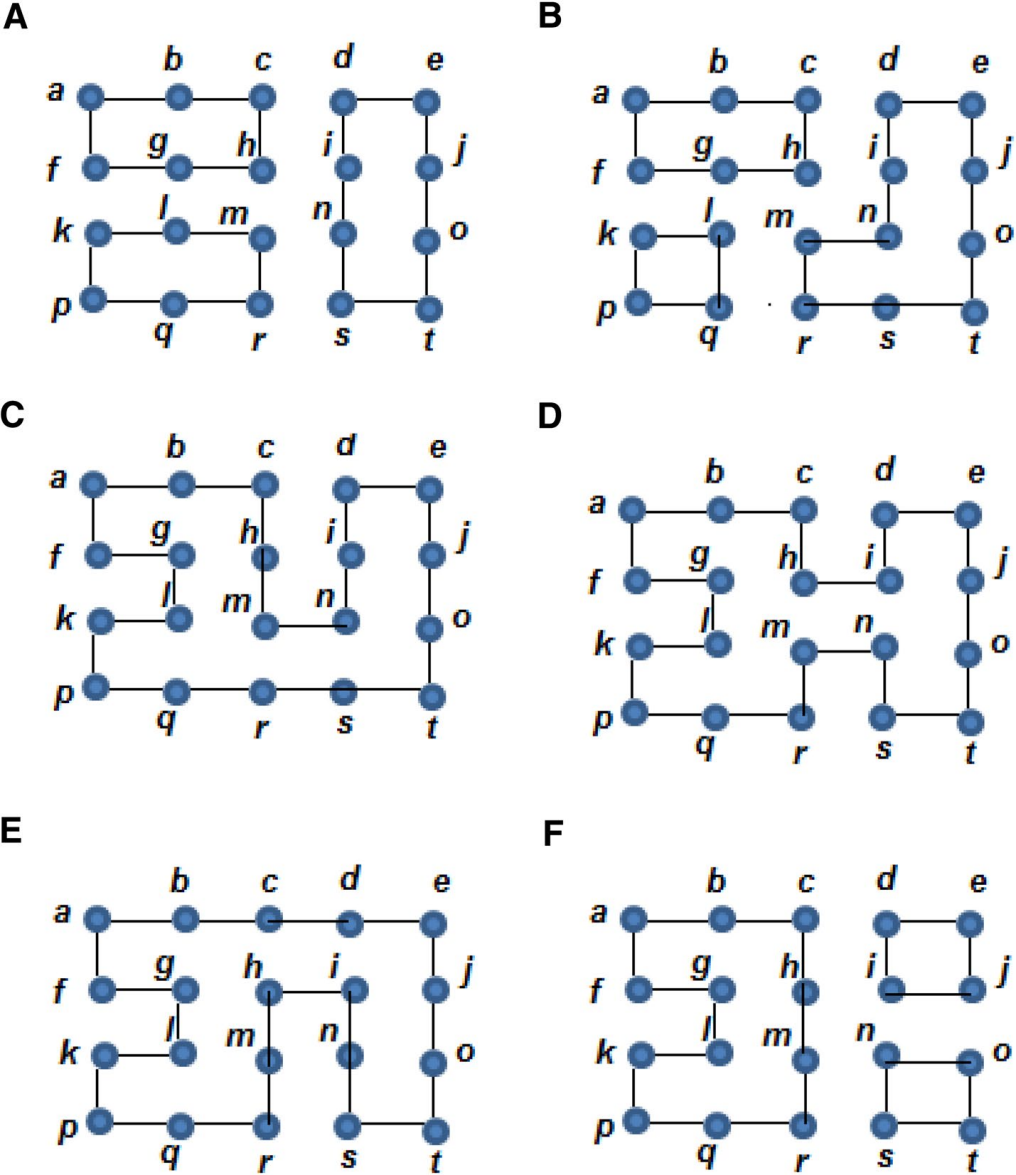
Producing n-factor graphs. **A** Reflecting C_1_ produces, **B** reflecting C_2_ produces, **C** reflecting C_3_ produces, **D** reflecting C_4_ produces, **E** reflecting C_5_ produces, **F** reflecting C_6_ produces

Figure 4 shows the status of the graph in Fig. 3 after applying reflections to the connectors C_1_, C_2_,…, and C_6_.

Therefore, if in a factorized representation of a graph there exists *m* different connectors, then to determine whether the graph is Hamiltonian or not, at most 2 ^*m*^ situations of the connectors must be tested. Knowing that each connector *C* has two situations *C* and *C*^−*1*^, each situation is called a switch.

For a certain graph, HC doesn’t have to be unique, and this is equivalent to the affirmation that the connectors that produce HC do not have to be unique. Distinctively to the Euler’s cycle (in which the determination of the direction of moving to pass each edge exactly one time is required), in the case of the Hamilton problem, the right edges that lead to pass each vertex once must be selected.

The previous arguments suggest that the idea of factorized representation, and the idea of connectors, will lead together to find the desired Hamilton cycle. Consequently, based on Theorem 1, starting with some *n*-factor representation (if possible, otherwise the graph is not Hamiltonian), the target is to make *n* = *1*. In other words, the scope is to join the *n* components by using some of the connectors.

Based on these aspects, a set of illustrative cases are presented below.

### *Example 4*

**Fig. 5:**
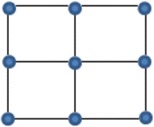
Not n-factor graph

The graph in Fig. 5 is not Hamiltonian because it is not *n*-factor graph.

### *Example 5*

**Fig. 6:**
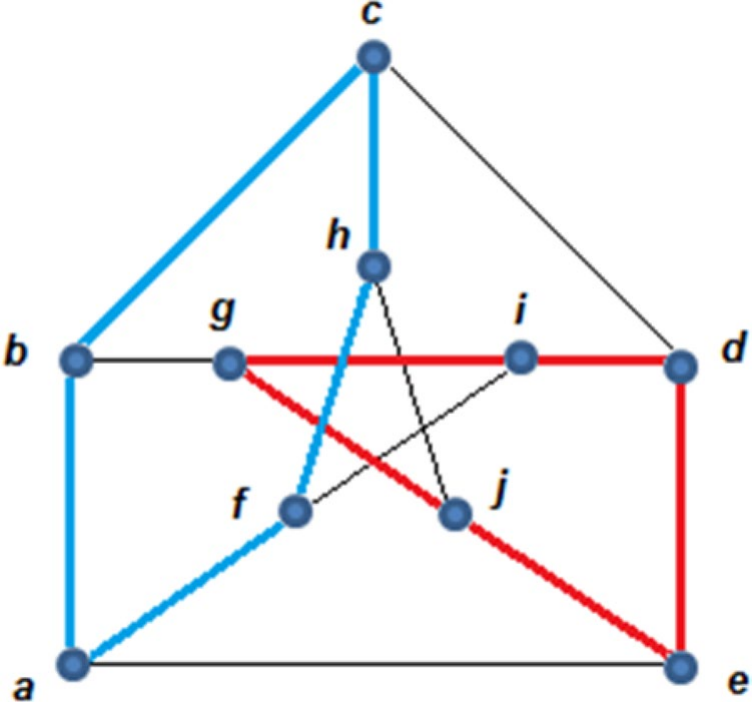
Petersen graph

**Fig. 7:**
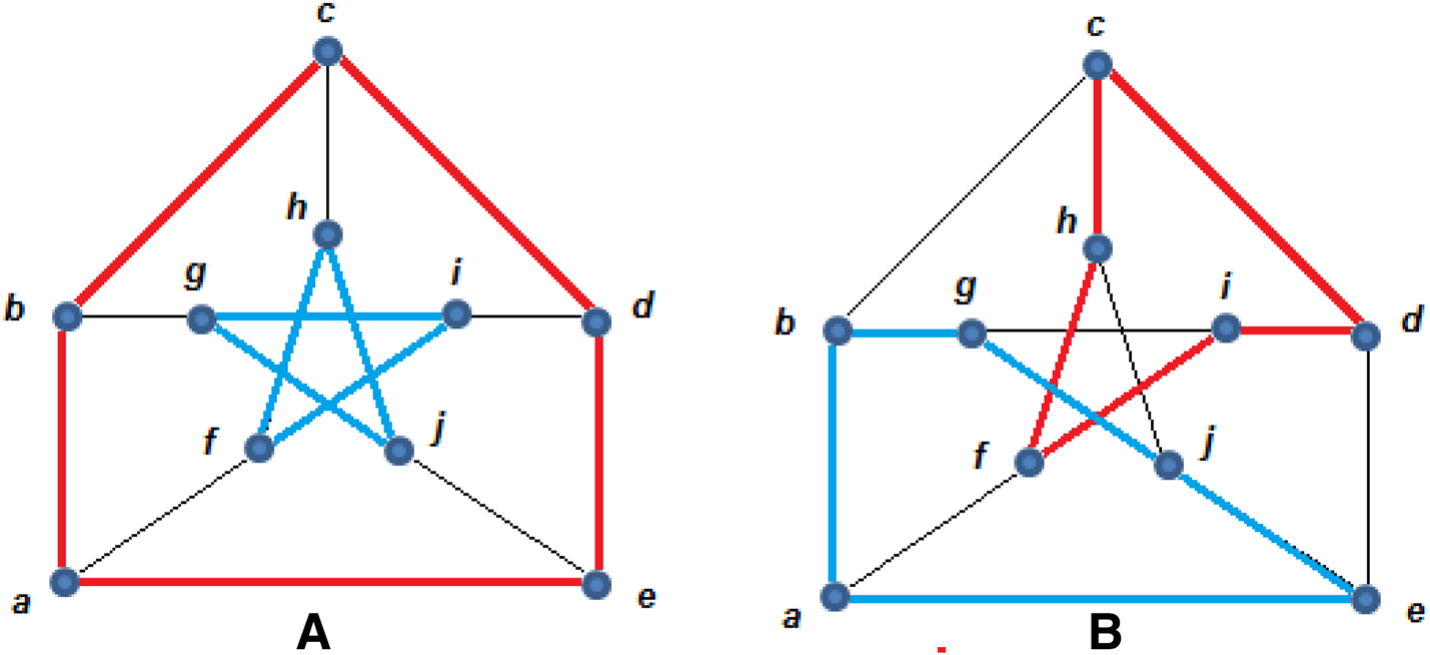
Connectors reflection of the Petersen graph from Example 5. **A** When connector C_1_: (*a*, *f*, *i*, *d*, *c*, *h*, *j*, *e*, *a*) is reflected and **B** connector C_2_: (*a*, *f*, *i*, *g*, *b*, *c*, *d*, *e*, *a*) is reflected

The Petersen graph as in Figs. 6 and 7.

In Example 5 it can be observed that the factorized representation of the Petersen graph is {G_1_, G_2_} where G_1_ is the red plot, and G_2_ is the blue plot. The components of the representation cannot be joined because reflecting any of the connectors will transfer the representation into another representation of two components; hence the Petersen graph is not Hamiltonian, although it is *n*-factor graph. In order for the reader to have a better understanding of this aspect, we will reflect all connectors.

## Results and discussion

In order to evaluate the proposed algorithms, their implementation was performed. The IDE used was Eclipse with JRE 7 and 1.6.0_37 JDK in combination with JGraph and JGraphT (open source java class libraries for the interface design) (http://www.jgraph.com, http://www.jgrapht.org). The specifications of the machine used in the experiments are 2 GB of RAM and 2.2 Ghz CPU speed with iOS version 10.8 as an operating system.

**Fig. 8:**
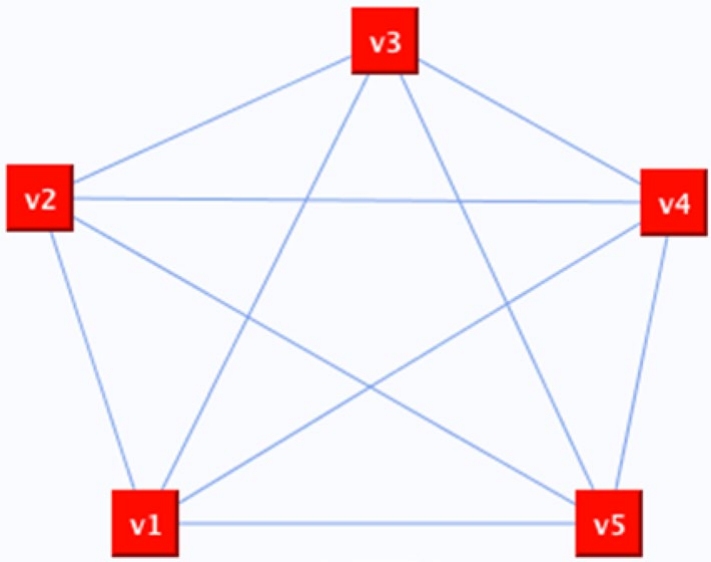
Complete graph

**Fig. 9:**
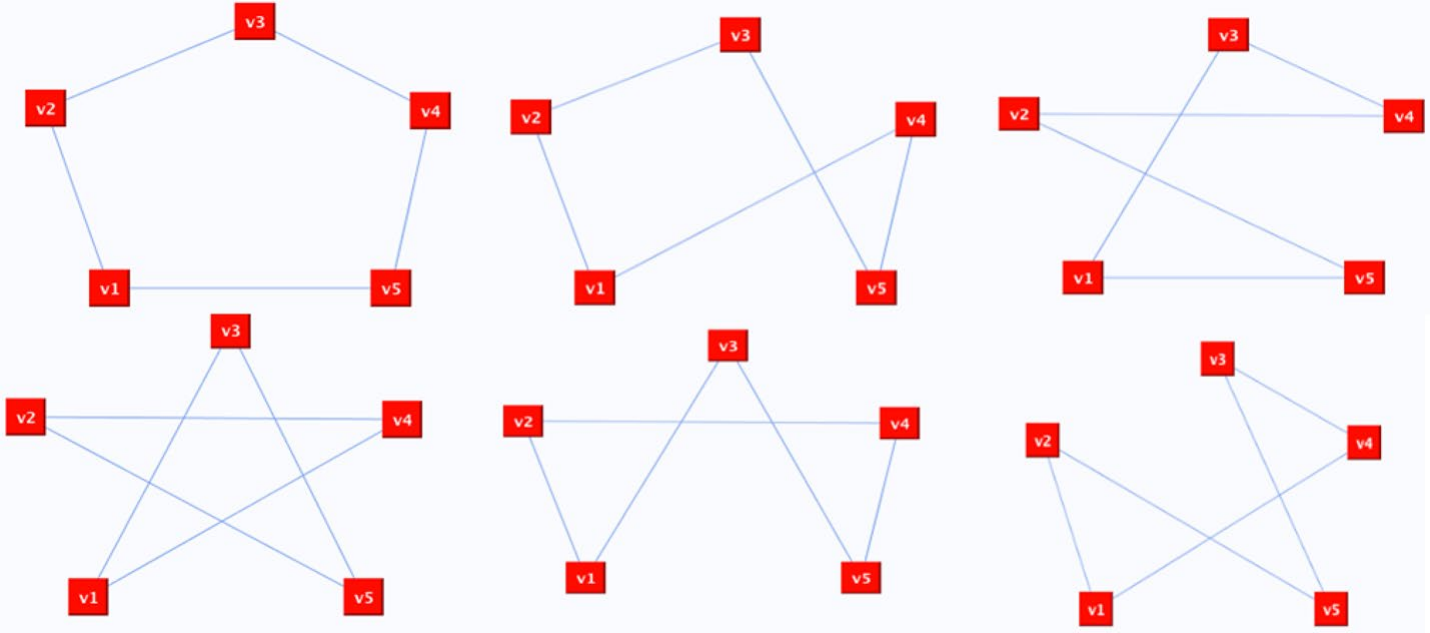
Different possible Hamiltonian circuits of the complete graph

The first example uses a complete graph of 5 nodes shown in Fig. 8. The Hamiltonian cycles of the complete graph shown in Fig. 9 are different because, in order to obtain the HC, the first step of the proposed methodology consists in randomly removing the edges incident with vertices of degree greater than 2.

**Fig. 10:**
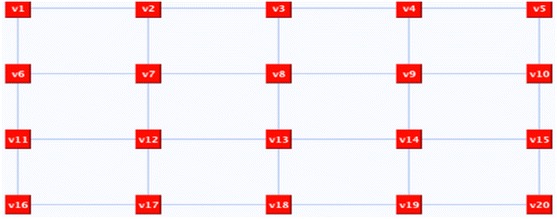
The input graph

**Fig. 11:**
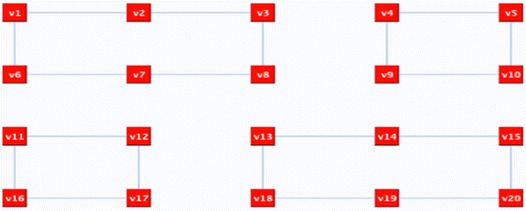
*n*-factor graph with *n* = 3

**Fig. 12:**
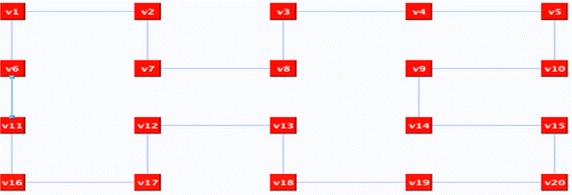
The graph after implementing a connector path

In some situations, this step is not enough and further refinements are required. Take, for instance, the 20 nodes graph in Fig. 10 as an example. The random deletion of surplus edges resulted in *n*-factor graph as shown in Fig. 11, which is a graph of disconnected cycles. In order to join the graph’s components, connector(s) need to be reflected. A connector is an even alternate edge path that is a cycle from a vertex to itself. One possible connector would be *C*: v6, X, v11, O, v12, X, v13, O, v14, X, v9, O, v4, X, v3, O v2, X, v7, O, v6. If we reflect *C*, the representation components will be joined and the Hamiltonian cycle will be revealed as illustrated in Fig. 12.

**Fig. 13:**
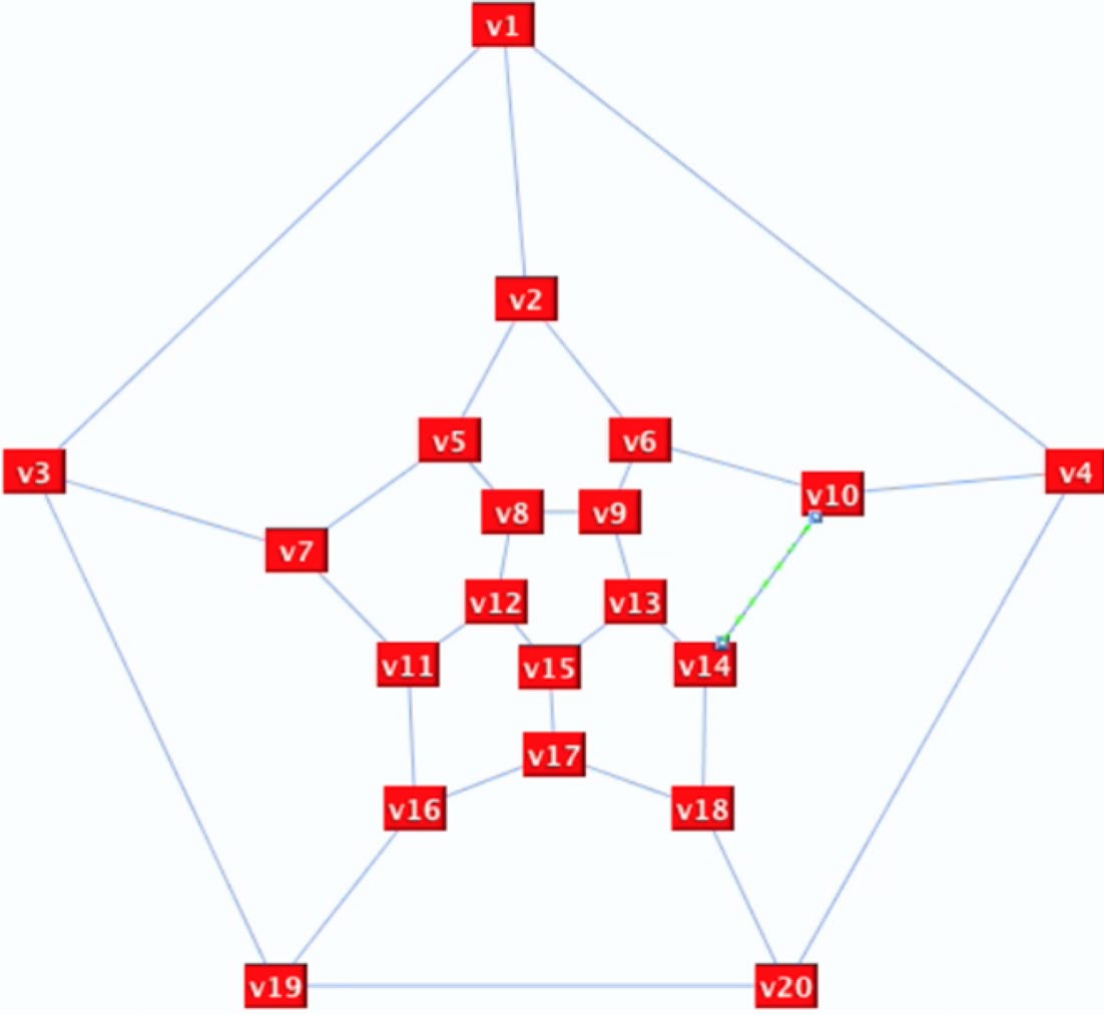
Dodecahedron graph

**Fig. 14:**
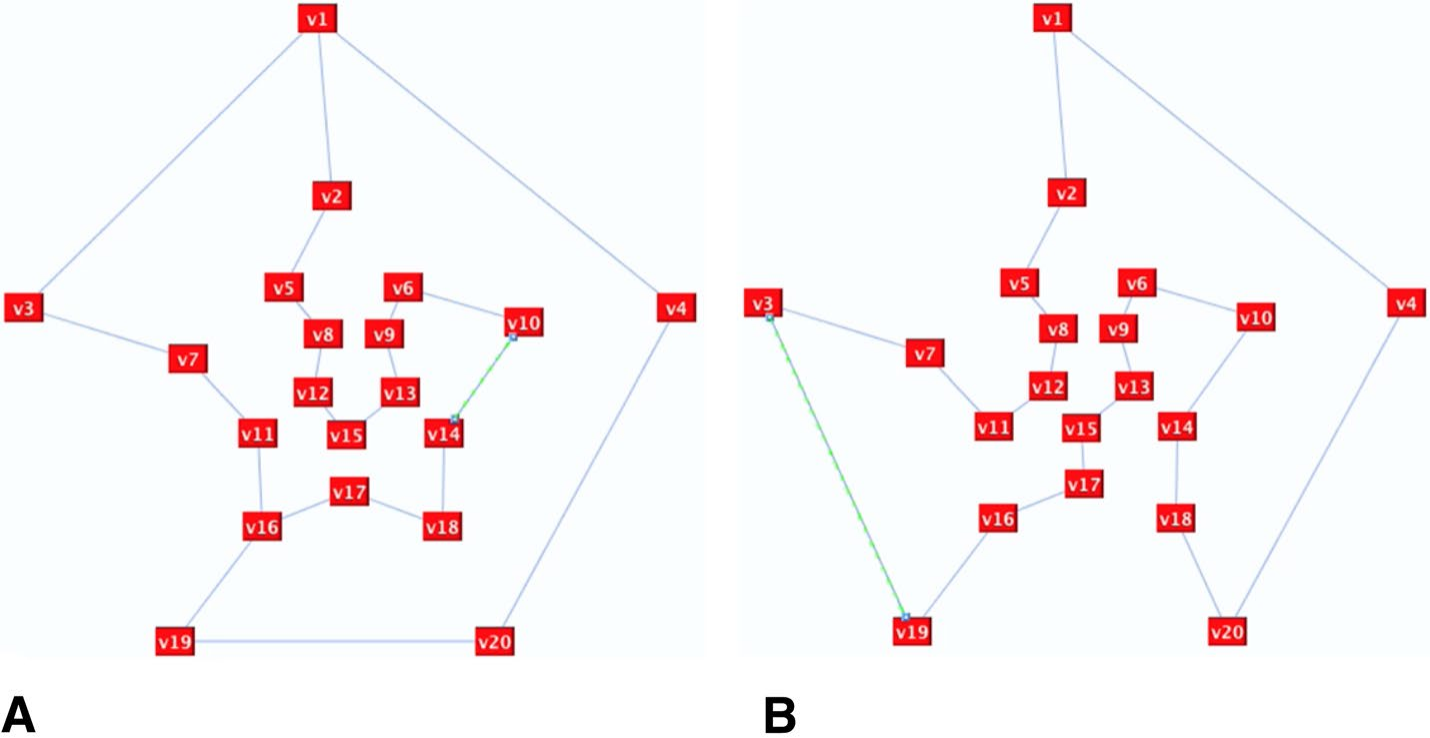
Dodecahedron graph after running the algorithms. **a** *S*_*D*_ = {v1, v16}, **b** the graph after implementing the destroyer

Figure 13 presents the Dodecahedron graph before implementing the algorithm. After performing a random deletion, the remaining set of our deletion is *S*_*D*_ = {v1, v16}, as shown in Fig. 14A, which shows a destroyer is needed to get rid of the extra edges; a destroyer is an odd alternate edge path between the two vertices of the remaining set. The path P: v1, O, v3, X, v19, O, v20, X, v18, O, v17, X, v15, O, v12, X, v11, O, v16 is a destroyer path. Figure 14B displays the Hamiltonian cycle of the Dodecahedron graph after the inverse of the destroyer P was reflected.

**Fig. 15:**
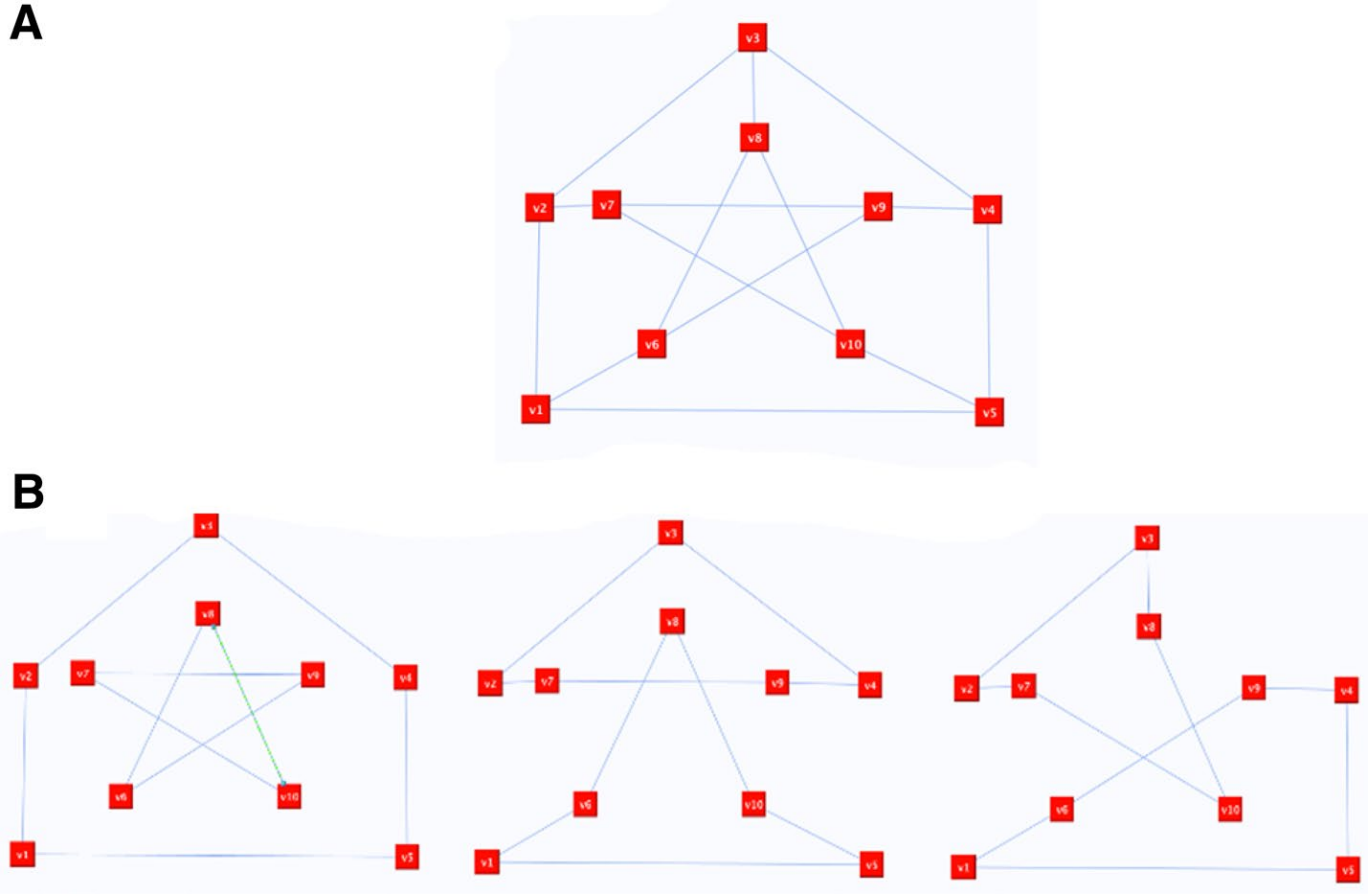
Petersen graph and some of its *n*-factor representations. **a** Peterson graph, **b** baby Hamiltonian representations of the Petersen graph

The next example is the Petersen graph, as shown in Fig. 15. After removing the extra edges from the Petersen graph, an *n*-factor graph was obtained. All connector path attempts failed to connect the graph and result in another *n*-factor graph. Some of these attempts are shown in Fig. 15B. Therefore, the graph is not Hamiltonian.

## Conclusion

As the existing solutions relate to the HC problem, this paper proposed a precise mathematical procedure for determining HC. Based on this procedure, an algorithm has been proposed and successfully implemented. The simulations results indicated that the algorithm works as expected. The graphs were visualized using a Java Applet with the aid of the JGraph library, where the steps of the algorithm are shown. Several graphs have been tested, and in all the cases, the algorithm provided successful results.

One of the possibilities of algorithm improvement include adding more constraints to the destroyer and connector functions, thus further reducing the search time. Also, computing the complexity of the algorithm and testing the algorithm on a variety of extensive graphs, needs to be implemented for future improvement.
